# *Plasmodium berghei* leucine-rich repeat protein 1 downregulates protein phosphatase 1 activity and is required for efficient oocyst development

**DOI:** 10.1098/rsob.220015

**Published:** 2022-08-03

**Authors:** Aline Fréville, Bénédicte Gnangnon, Annie Z. Tremp, Caroline De Witte, Katia Cailliau, Alain Martoriati, El Moukthar Aliouat, Priyanka Fernandes, Cerina Chhuon, Olivier Silvie, Sabrina Marion, Ida Chiara Guerrera, Johannes T. Dessens, Christine Pierrot, Jamal Khalife

**Affiliations:** ^1^ Univ. Lille, CNRS, Inserm, CHU Lille, Institut Pasteur de Lille, U1019—UMR 9017—CIIL—Centre d'Infection et d'Immunité de Lille, 59000 Lille, France; ^2^ Department of Infection Biology, Faculty of Infectious and Tropical Diseases, London School of Tropical Medicine and Hygiene, Keppel Street, WC1E 7HT London, UK; ^3^ Univ. Lille, CNRS, UMR 8576-UGSF-Unité de Glycobiologie Structurale et Fonctionnelle, F-59000 Lille, France; ^4^ Sorbonne Université, INSERM, CNRS, Centre d'Immunologie et des Maladies Infectieuses, F-75013 Paris, France; ^5^ Proteomics platform 3P5-Necker, Université Paris Descartes - Structure Fédérative de Recherche Necker, INSERM US24/CNRS UMS3633, Paris, France

**Keywords:** leucine-rich repeat protein 1, SDS22, protein phosphatase 1, PP1, inhibitor 3

## Abstract

Protein phosphatase 1 (PP1) is a key enzyme for *Plasmodium* development. However, the detailed mechanisms underlying its regulation remain to be deciphered. Here, we report the functional characterization of the *Plasmodium berghei* leucine-rich repeat protein 1 (PbLRR1), an orthologue of SDS22, one of the most ancient and conserved PP1 interactors. Our study shows that PbLRR1 is expressed during intra-erythrocytic development of the parasite, and up to the zygote stage in mosquitoes. PbLRR1 can be found in complex with PbPP1 in both asexual and sexual stages and inhibits its phosphatase activity. Genetic analysis demonstrates that PbLRR1 depletion adversely affects the development of oocysts. PbLRR1 interactome analysis associated with phospho-proteomics studies identifies several novel putative PbLRR1/PbPP1 partners. Some of these partners have previously been characterized as essential for the parasite sexual development. Interestingly, and for the first time, Inhibitor 3 (I3), a well-known and direct interactant of *Plasmodium* PP1, was found to be drastically hypophosphorylated in PbLRR1-depleted parasites. These data, along with the detection of I3 with PP1 in the LRR1 interactome, strongly suggest that the phosphorylation status of PbI3 is under the control of the PP1–LRR1 complex and could contribute (in)directly to oocyst development. This study provides new insights into previously unrecognized PbPP1 fine regulation of *Plasmodium* oocyst development through its interaction with PbLRR1.

## Graphical abstract

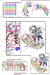

## 1. Introduction

The causative agent of malaria, a protozoan of the genus *Plasmodium*, displays a complex life cycle involving diverse host environments and extensive variations in parasite shape, size and motility, along with a remarkably rapid mode of cell proliferation [1–3]. Recent studies have highlighted the pivotal role of protein phosphorylation as a major regulatory mechanism used by the parasite to quickly respond to environmental changes and sustain its development. Genome-wide kinome and phosphatome functional screens associated with chemical genetic approaches led to the identification of pathways essential to the progression of *Plasmodium* at every stage of its development, cell division or parasite–host interactions. The modulation of the host phospho-signalling pathway is also now established as a pivotal feature of the infection [4–11].

*Plasmodium* protein phosphatase type 1 (or PP1) is an enzyme of considerable interest as it seems to be responsible for most of the dephosphorylation processes in the parasite [12]. Biochemical studies using inhibitory compounds of the catalytic subunit (PP1c), such as calyculin A or okadaic acid, suggested a crucial role of PP1 in *Plasmodium falciparum* intra-erythrocytic development [12–14]. During the final stages of intra-erythrocytic development, PfPP1 seems to modify the phosphorylation status of the skeleton-binding protein 1 (PfSBP1) and to be implicated in the release of infectious merozoites [15]. These early findings are consistent with more recent reverse genetic analyses which demonstrate PfPP1c essentiality for blood-stage parasite development and egress from erythrocytes [7,16,17].

Despite its biological significance, little is known about the mechanisms regulating *Plasmodium* PP1c activity. One of the key regulatory mechanisms of this class of enzymes is through its interaction with numerous partners (PIPs: PP1c-interacting proteins). Various PIPs (regulators and/or substrates of PP1) bind to PfPP1c to create various protein complexes which are each involved in diverse specific cellular functions [18–21]. In a continuing effort to identify *Plasmodium* PP1c regulators and to decipher the signalling pathways regulated by PP1, an examination of the *P. falciparum* PP1c interactome has led to the identification of more than 134 interactors [22]. Among these, four conserved regulators (Pf-inhibitor 2 (PfI2), Pf-inhibitor 3 (PfI3), Pfeif2*β* and PfLRR1) have been examined for their essentiality during blood-stage development and ability to regulate PP1c activity [23–27]. Interfering peptides designed to disrupt PfPP1 complexes have been proved efficient in inhibiting parasite growth *in vitro*, paving the way for strategies using PP1c and PIPs as therapeutic targets [24,28–30]. More recently, studies have focused on three PIPs restricted to the apicomplexan genus. The first one, PbGEXP15, seems to be involved with PP1c in mRNA splicing and proteasome pathways. Its disruption leads to the generation of attenuated parasites with effects on both asexual and sexual development [31]. The second, RCC-PIP, a kinase anchoring protein, is involved in PP1c regulation and seems essential for *P. falciparum* erythrocytic development [32]. Finally, a pseudo tyrosine kinase-like named PbpTKL was found to be exported to the host cell and to interact with proteins involved in cytoskeleton organization, erythrocyte maturation and homeostasis [33].

Despite the biological diversity of PP1 holoenzymes, around 85% of PIPs share a canonical RVXF binding motif critical for the interaction with PP1c and the regulation of its functions [34–36]. However, leucine-rich repeat protein 1 (LRR1), one of the major and most ancient PIPs, lacks this motif and instead relies on leucine-rich repeats domains to bind PP1c [37]. LRR1 is an orthologue of human SDS22 and was first described in yeast as a regulator of cell division, more precisely in the metaphase-to-anaphase progression [38–40]. During anaphase, SDS22 is required for the stabilization of the kinetochore-spindle attachment [41,42]. SDS22 specifically defines PP1c localization at the kinetochore, and thereby phosphorylation status of Aurora B, a major actor of cell division involved in cytokinesis, chromosome condensation and activation of the spindle assembly checkpoint [43–46]. In a recent crystallography study, Heroes *et al.* revealed that human SDS22 most likely interacts with PP1c via a domain relatively remote from binding sites of previously described interactors. They speculated that SDS22 could act as the third interactor of multiple PP1 complexes, thereby exhibiting diverse functions [47]. In addition to cell division, SDS22 has been implicated in multiple processes such as cell shape and polarity [48], epithelium integrity [49], sperm motility [50,51] and plant immunity [52–55], and is now receiving increasing interest as a therapeutic target in cancer studies [56,57].

In *P. falciparum,* LRR1 interacts with PP1c via several LRR domains and unexpectedly via its C-terminal LRR cap motif [30]. Additionally, PfLRR1 inhibits PP1c activity [23]. Genome-wide saturation mutagenesis revealed that the gene is non-mutable and therefore probably essential to *P. falciparum* blood-stage completion [58]. Its precise involvement at this stage of the life cycle remains to be addressed.

Moreover, nothing is known about *Plasmodium* LRR1 function during development in the mosquito. To address this knowledge gap, we performed a functional study of LRR1 in relation to its interaction with PP1c in *P. berghei*. We demonstrate that PbLRR1, like its *P. falciparum* counterpart, interacts with PP1c and downregulates its activity. However, unlike PfLRR1, PbLRR1 can be knocked-out without obvious impact on the parasite's intra-erythrocytic development. Using a combination of reverse genetics followed by interactome and phospho-proteomic analyses, we demonstrate that PbLRR1 influences oocyst development in the mosquito and, likely via its PP1c regulatory activity, is involved in the phospho-regulation of proteins essential for sporogonic development.

## 2. Material and methods

### 2.1. Ethics statement for animal experimentation

All laboratory animal work was carried out in accordance with European Directive 2010/63/EU for animal experiments and approved by local animal welfare ethics review bodies. Experiments were generally conducted in pathogen-free 6–8 weeks old male CD1 mice, maintained in filter cages. Animal welfare was assessed daily, and animals were humanely killed upon reaching scientific or clinical endpoints. Mice were infected with parasites resuspended in phosphate-buffered saline by intraperitoneal injection, or by infected mosquito bite on anaesthetized animals. Intra-erythrocytic parasitemia was monitored regularly by collecting a small volume of blood from a superficial tail vein. Drugs were administered by intraperitoneal injection or when possible were supplied in drinking water. Parasitized blood was harvested by cardiac bleed under general anaesthesia without recovery.

### 2.2. Plasmids, antibodies and primers

Commercial plasmid pGADT7 was purchased from Clontech while pbGEM-232294, PL1886 and pG362 were kindly gifted from the Plasmogem-Wellcome Sanger Institute (Cambridge, UK), Dr B. Franke-Fayard (Leiden University Medical Center, The Netherlands) and Dr N. Philip (The University of Edinburgh, UK), respectively. The antibodies used in this study were: rat anti-HA mAb (Sigma, 11867423001), rabbit anti-RFP pAb (MBL, PM005), mouse anti-HA mAb (Roche, (clone 12CA5, 1158381600) and mouse anti-PP1 mAb (SantaCruz Biotechnology). The polyclonal antibodies raised against *Toxoplasma* actin and the polyclonal antibody raised against *Plasmodium falciparum* LRR1 were already available in the laboratory [23,59]. All the primers used in this study are listed in electronic supplementary material, table S1.

### 2.3. Bioinformatic analysis of PbLRR1 structure and its binding mode to PbPP1

PbLRR1 protein sequence was retrieved from PlasmoDB (PBANKA_0516600) after OrthoMCL (https://orthomcl.org/orthomcl) analysis of PfLRR1 (PF3D7_1032800). Leucin Rich Repeat (LRR) domains were predicted using Prosite (https://prosite.expasy.org/) and aligned using MAFFT (https://www.ebi.ac.uk/Tools/msa/mafft [60]) to highlight the LRR consensus sequence. Pictures of PbLRR1 tertiary structure pictures were made using UCSF Chimera 1.14 (https://www.cgl.ucsf.edu/chimera [61]).

In order to make a prediction of the PbLRR1/PbPP1 complex structure, an *in silico* docking was performed using HADDOCK 2.2 server (https://milou.science.uu.nl/services/HADDOCK2.2/haddock.php [62]). The tertiary structure of *P*bLRR1 was predicted using I-Tasser (https://zhanglab.ccmb.med.umich.edu/I-TASSER [63]) and the tertiary structure of PbPP1 was obtained from SwissModel (https://swissmodel.expasy.org/repository/uniprot/A0A509AL46 [64]). To inform the docking server, residues potentially involved in binding were obtained from Heroes *et al.* for HsSDS22 and HsPP1c [47]. After protein sequence alignment using MAFFT, the corresponding residues were inferred in PbLRR1 (D87, Y109, E131, W153, D221, E241, W243, Y268) and PbPP1c (K145, K148) respectively (electronic supplementary material, figure S1B and S1C). Upon docking, 10 clusters of protein complexes were returned (electronic supplementary material, table S2, score docking). The best model of each cluster was saved for further analysis. These 10 models were then superimposed using UCSF Chimera 1.14 to highlight the diverse PbLRR1/PbPP1c binding modes returned by the docking. The best-ranked cluster presenting the most common binding mode (5 clusters out of 10 presented this binding mode here) was kept for further analysis. Hydrogen bonds (H-bonds) were predicted for the 4 best models within this cluster using UCSF Chimera (FindHBind tool, H-bond relaxation: 0.4 Å and 60°) and were confirmed using PIC (http://pic.mbu.iisc.ernet.in/index.html [65]). The model displaying the most conserved H-bond configuration among all models was focused on for further binding analysis and is displayed on the main text figures. All pictures of protein complexes (with and without H-bonds) were made using UCSF Chimera 1.14.

### 2.4. Generation of transgenic parasites

PbLRR1 KO lines were generated by double homologous recombination of a NotI-linearized PlasmoGEM vector (PbGEM-232294, Wellcome Sanger Institute, [66]) transfected into PbGFP ANKA parasites [67].

A C-terminal AID-HA-tagged PbLRR1 line was generated by single homologous recombination. A 1150-bp region of PbLRR1 starting 289 bp downstream from the start codon and lacking a stop codon (primers Pr2 and Pr3, electronic supplementary material, table S1) was inserted into pG362 vector [68] and BsaBI-linearized before transfection into an OsTIR1 expressing pG230 *P. berghei* ANKA line (both plasmid and parasites are gifts from N. Philip, University of Edinburgh, UK).

Similarly, a C-terminal mCherry endogenously tagged PbLRR1 was generated by single homologous recombination of a 1025 bp region of PbLRR1 lacking the stop codon and starting 433 bp downstream from the start codon (Pr13-Pr14, electronic supplementary material, table S1) inserted into PL1886 vector and Spe1-linearized before transfection into PbGFP ANKA strain [67].

Transfections were carried out as previously described [69]. Briefly, Nycodenz-enriched schizonts were electroporated with 5–10 µg linearized DNA and immediately IV-injected into naive mice. From day 1 post-infection, transfectants were selected with pyrimethamine (10 mg l^−1^ (Sigma-Aldrich) in drinking water) as all vectors contain a dhfr/ts expression cassette (electronic supplementary material, figures S2–S4). Regarding the C-terminal AID-HA tagged line, the drug selection is associated with the expression of a GFP fluorescent marker, as their expression are both driven by the bidirectional *pbeef1α* promoter [68]. Drug selection was carried out by repeated passages in mice. Transgenic parasites were cloned by limiting dilution.

Knock-out parasites were genotyped using diagnostic PCR (electronic supplementary material, figure S3B and S3C). Integration and deletion were confirmed using primers Pr4/Pr5 (5′ integration), Pr6/Pr7 (3′ integration) and Pr1/Pr2 (deletion). Additionally, Knock-out parasite transcription status was finally determined by RT-PCR (electronic supplementary material, figure S3D).

Integration in the C-terminal AID-HA and mCherry tagged PbLRR1 lines were checked using primers Pr1/Pr8 (AID-HA 5′integration), Pr9/Pr10 (AID-HA 3′integration) (electronic supplementary material, figure S2B), Pr1/Pr15 (mCherry integration) (electronic supplementary material, figure S4B). The correct size expression of PbLRR1-AID-HA and PbLRR1-mCherry proteins were confirmed by western blot analysis. Regarding the PbLRR1-AID-HA line, the degradation of the fused protein in the presence of 3-indoleacetic acid (IAA, Sigma-Aldrich), 0.5 µM for 30 min at 37°C) was used as a control (electronic supplementary material, figure S2C). Regarding the PbLRR1-mCherry line, parental strain was used as control (electronic supplementary material, figure S4C). All samples were boiled in Laemmli buffer and electrophoresed on a 4–12% SDS-polyacrylamide gel. Proteins were subsequently transferred onto nitrocellulose membranes (Amersham Biosciences). Membranes were probed with rat anti-HA mAb (1 : 500), rabbit anti-RFP pAb (1 : 2000), polyclonal anti-Tg Actine (1 : 1000, loading control) as primary antibodies and IgG-HRP (1 : 2000, Sigma-Aldrich) as secondary antibodies. Membranes were developed using chemiluminescence detection (SuperSignal West Dura Extended Duration Substrate, Life Technologies) according to the manufacturer's instructions.

### 2.5. Isolation of parasites stages

Parasite stages were isolated as previously described [70,71].

Asexual stages: briefly, to obtain schizont stages, blood was collected by cardiac puncture from euthanized mice 3 days post-infection (p.i.) (parasitemia around 2–3%) and cultured in schizont medium (RPMI 1640 medium containing 25 mM HEPES, 0.4% Albumax, 0.2 mM hypoxanthine and 20 µg ml^−1^ gentamycin) at 37°C, 54 rpm for 20 h. Schizonts were purified on a 60% Nycodenz cushion (27.6% w/v Nycodenz in 5 mM Tris-HCl pH 7.20, 3 mM KCl, 0.3 mM EDTA) for 25 min at 270 g.

Non-activated and activated Gametocytes: mice were pre-treated with phenylhydrazine (0.2 ml of 6 mg ml^−1^ injected intraperitoneally) 3 days prior to infection. Day 3 p.i. mice were treated with sulfadiazine (Sigma) for 2 days (20 mg L^−1^ in drinking water). On day 5 p.i., blood was collected by cardiac puncture and gametocytes were purified on a 48% Nycodenz gradient in coelenterazine loading buffer ((CLB), containing PBS, 20 mM HEPES, 20 mM glucose, 4 mM sodium bicarbonate, 1 mM EGTA, 0.1% w/v bovine serum albumin, pH 7) for 10 min at 1082 *g*. When needed, gametocytes were activated in ookinete medium (RPMI1640 containing 25 mM HEPES and 10% fetal calf serum, pH 8) for 15 min at 20°C.

Zygotes: blood cells from day 5 p.i. mice were placed in ookinete medium for 3 h at 20°C and zygotes were purified on a 12% nycodenz cushion for 15 min at 500 *g* [71].

For each stage of isolation, the purity of the parasite preparation was systematically checked by microscopic examination of Giemsa-stained smears.

### 2.6. Phenotypic analyses

Phenotypic analyses of the Knock-out lines were performed as previously described [72]. Briefly, asexual growth and gametocytemia were monitored every day (up to 7–8 days) using Giemsa-stained blood smears of phenylhydrazine pre-treated mice intraperitoneally injected with 10^6^ parasites. The number of nuclei per schizont was counted on Giemsa smears after *in vitro* maturation of trophozoites in RPMI 1640 medium for 24 h at 37°C. Gametocyte activation and ookinete conversion rates were examined *in vitro*. Male exflagellation was analysed on day 5–6 pi. Gametocyte-containing blood was mixed with ookinete culture medium (1 : 4 with RPMI1640 containing 25 mM HEPES and 10% fetal calf serum, pH 8) for 15 min at 20°C. Exflagellation centres were counted on 10–12 fields using light microscopy (63× objective and 10× ocular lens). The fertilization rate (ookinete conversion rate) is defined as the percentage of female gametes that develop into mature ookinetes determined by counting female gametes and mature ookinetes in Giemsa-stained blood smears 16–18 h after *in vitro* induction of gamete formation [73,74].

Mosquito transmission experiments were performed as previously described [75]. Briefly, around 70 female *Anopheles stephensi* mosquitoes (aged 4 to 6 days) were fed for 20 min on anaesthetized, parasite-infected mice. At day 9 and day 14 post-infection, mosquito midguts were dissected, and oocysts were counted and measured using a 40× objective and eyepiece graticule on an Olympus CX43 microscope. If desired, salivary glands were dissected at 21 days post-infection, pooled and sporozoites released by gentle homogenization, followed by sporozoite counts using a haemocytometer. Mosquitoes infected with WT or PbLRR1 KO parasites were used to transmit parasites to mice in bite-back experiments. Blood stage parasitemia was measured by light microscopy of Giemsa-stained blood films.

*In vitro* sporozoite infection assays were performed as previously described by Langlois *et al.* [76]. HepG2 cells were seeded in 96 well plates (3 × 10^4^ per well on day prior experiment) and incubated for 3 h with 10 000 sporozoites collected from the salivary glands of infected mosquitoes 21–28 days post-feeding. After 24 and 48 h, cultures were fixed and the number of GFP-positive exo-erythrocytic forms (EEFs) in triplicate wells was determined by fluorescence microscopy.

For *in vivo* sporozoite infection assays, salivary gland sporozoites were collected from infected mosquitoes 21–28 days post-feeding and I.V. injected in C57BL/6 mice. Parasitemia was monitored every day up to 7 days by flow cytometry on a Guava EasyCyte 6/2 L bench cytometer (Millipore).

### 2.7. RNA extraction and quantitative RT-PCR

Activated gametocytes were purified as described above, pelleted and immediately frozen in Trizol reagent (15596018, Invitrogen) prior to RNA extraction. RNA was isolated according to manufacturer's instructions (Invitrogen) and treated with DNase I. Treated RNA (500 ng to 1 µg) was then used in reverse transcription reactions (SuperScript III Reverse Transcription kit, Invitrogen) following the manufacturer's instructions. *Pblrr1* gene expression analysis in WT and KO mutant was performed using SYBR Selected Master Mix (Life Technologies) according to manufacturer's instructions. 3 µM of each primer and around 10 ng of cDNA were diluted in nuclease-free water in a 20 µl final volume. Analysis was performed on the Mx3000P system (Agilent Technologies) using the following cycling conditions: 1 cycle of 50°C for 2 min, 1 cycle of 95°C for 10 min, 40 cycles of 95°C for 30 s, 60°C for 30 s before fluorescence detection followed by a dissociation curve determined with 1 cycle of 95°C for 1 min, 55°C for 30 s and 95°C for 30 s. Three technical replicates and two biological replicates were performed. All primers were designed using Primers3 + software program (https://primer3plus.com/cgi-bin/dev/primer3plus.cgi) and are listed in electronic supplementary material, table S1.

### 2.8. Immunofluorescence assay

Parasite stages were collected as described above and fixed (4% paraformaldehyde in PBS) for 10 min at RT. Parasite smears were prepared on poly-L-lysine adhesion slides (Thermofisher) and air-dried. The cells were then permeabilized (0.5% triton X-100 in PBS) for 10 min prior to blocking (3% BSA in PBS) for 1 h at RT. Parasites were incubated with rat anti-HA mAb (1 : 200 in PBS, 1% BSA) for 1 h at RT. Subsequently, Alexa 594-conjugated anti-rat IgG (Invitrogen, 1 : 2000 in 1 %BSA-PBS) were used as secondary Ab in addition to DAPI (1 µg ml^−1^) for 1 h at 37°C. Slides were mounted in Mowiol. Confocal imaging was performed using a 63× Plan Apochromat (1.4 NA) oil objective on the LSM880 confocal microscope (Zeiss) and processed using Zen Software.

### 2.9. *Xenopus* oocytes analysis

Capped mRNA (cRNA) encoding PbLRR1 was synthesized *in vitro* using a T7 mMessage mMachine kit (Ambion) and HindIII-linearized PbLRR1-pGADT7 as template. 60 nl (60 ng) of cRNA was injected in the equatorial region of stage VI *Xenopus laevis* oocytes (20 oocytes per assay removed from 2–3 animals) and incubated at 19°C in ND96 medium (96 mM NaCl, 2 mM KCl, 1 mM MgCl_2_, 1,8 mM CaCl_2_, 5 mM Hepes pH 7.4 supplemented with 50 µg ml^−1^ Streptomycin/Penicillin, 225 µg ml^−1^ sodium pyruvate, 30 µg ml^−1^ trypsin inhibitor). Regarding the peptide microinjection experiments, 100 ng of peptides were pre-injected into oocytes 1 h prior the injection of 100 ng of cRNA as previously described in [28]. After 15 h, the germinal vesicle breakdown (GVBD) was detected by the appearance of a white spot at the centre of the animal pole [77]. The injection of progesterone (PG), PBS and unrelated cRNA control (obtained from a previous construct: Human EGF [78]) was used as control. Expression of PbLRR1 and interaction with XePP1 was confirmed by immunoprecipitation assay and western-blotting analysis. Following 15 h of expression, 25 oocytes were lysed in 250 µl of buffer A (50 mM Hepes pH 7.4, 500 mM NaCl, 0.05% SDS, 5 mM MgCl2, 1 mg ml^−1^ bovine serum albumin, 10 µg ml^−1^ leupeptin, 10 µg ml^−1^ aprotinin, 10 µg ml^−1^ soybean trypsin inhibitor, 10 µg ml^−1^ benzamidine, 1 mM PMSF, 1 mM sodium vanadate) and centrifuged at 4°C for 15 min at 10 000 *g*. Supernatants were incubated for 1 h 30 at 4°C with anti-PP1 antibodies (1/200) in the presence of protein A-sepharose beads (20 µl of 50% bead slurry, Sigma) for 1 h 30 at 4°C under gentle rocking. After a brief centrifugation and 3 rinses with buffer A, immune complexes were eluted with 25 µl of Laemmli sample buffer (Biorad) and heated at 96°C for 3 min. For electrophoresis, proteins (15 µl of each immune complex) were separated by 4–20% SDS-PAGE gels (mini protean TGX, BioRad), for 1 h at 200 V in denaturing buffer (0.1% SDS; 0.3% TRIS base; 1.44% glycine), and transferred onto a nitrocellulose membrane (Amersham Hybond, Dutscher, Bernolsheim, France) by wet transfer (0.32% TRIS; 1.8% glycine; 20% methanol, Sigma Aldrich) for 1 h at 100 V. For western blot, membranes were blocked with 5% low fat dry milk in TBS added with 0.05% Tween (SA), and incubated overnight at 4°C with specific primary anti-PP1 (1 : 1500) or anti-HA (1 : 1500) antibodies. Membranes were incubated 1 h at room temperature with secondary antibody (1 : 10000, Trueblot, eBiosciences) raised against native IgG to avoid recognition of denatured light and heavy IgG chains. After three washes in TBS-Tween for 10 min, the signals were detected and developed using the ECL Select detection system (Amersham) on hyperfilm (MP, Amersham).

### 2.10. Immunoprecipitation and mass spectrometry

Experiments were carried out as previously described [31]. Purified schizonts, gametocytes or zygotes obtained from PbPP1c-mCherry, PbLRR1-mCherry and parental wild-type strains (control) were suspended in lysis buffer (50 mM Tris, 0.5% Triton X100 and protease inhibitor cocktail (Roche), pH 8). After 10 freeze–thaw cycles, sonication (30″ ON/OFF cycles) and subsequent centrifugation (5 h, maximum speed, 4°C), the soluble fraction was used for immunoprecipitation with RFP-Trap1_A beads (Chromotek). Beads were equilibrated with dilution buffer (20 mM Tris, 150 mM NaCl, 0.5% Triton X-100 and protease inhibitor cocktail (Roche), pH 7.5) and subsequently put in contact with parasite soluble fraction overnight at 4°C on a rotating wheel. After centrifugation (2500 g for 2 min), the beads were washed 10 times with dilution buffer and proteins eluted in Laemmli buffer. Western-blot analysis was performed using anti-RFP pAb (1 : 2000) as primary antibody followed by goat anti-rabbit IgG-HRP (1 : 20 000). Then, membranes were washed and probed with polyclonal anti-PfLRR1 (1 : 500) followed by Mouse TrueBlot1 Ultra: Anti-Mouse Ig HRP (1 : 20 000, eBioscience).

Immunoprecipitation eluates were supplemented with SDS to a final concentration of 5% for protein digestion. S-TrapTM micro spin column (Protifi, Farmingdale, NY, USA) digestion was performed on immunoprecipitation eluates according to manufacturer's protocol but using two extra washing steps for thorough SDS elimination. Samples were digested with 2 µg of trypsin (Promega) at 47°C for 2 h. After elution, peptides were vacuum dried down.

### 2.11. Sample preparation for proteome and phospho-proteome analysis

*Plasmodium berghei* zygotes were purified as described before and samples prepared as previously described [31]. Briefly, parasites were treated with 0.15% saponin-PBS buffer to remove host cell proteins. Then, parasite proteins were extracted using RIPA buffer (Pierce, 8 99 000), Halt Protease, Phosphatase Inhibitor Cocktail (Thermo Fisher Scientific) prior to DNase I (Thermo Fisher Scientific) treatment. Proteins were quantified using the Pierce BCA Protein Assay Kit (Life Technologies). 100 µg of proteins were reduced with tris(2-carboxyethyl) phosphine (0.05 M at room temperature for 10 min). MS sample preparation was performed using a FASP method (filter aided sample preparation) [79]. An average of 10 µg of the digested proteins was used for the analysis of total proteome. The remaining samples were used for phosphopeptide enrichments.

Phosphopeptide enrichment was carried out using a Titansphere TiO_2_ Spin tip (3 mg/200 µl, Titansphere PHOS-TiO, GL Sciences Inc, Japan) on 90 µg of digested proteins for each biological replicate according to manufacturer's instructions with modifications. Peptides were resuspended in 20 µl of 10% ACN%, 0.1% TFA, mixed with 100 µl of 75% acetonitrile, 0,075% TFA, 25% lactic acid before phosphopeptides adsorption to the TiO_2_. Phosphopeptides were eluted by the sequential addition of 50 µl of 5% NH4OH and 50 µl of 5% pyrrolidine. Phosphopeptides were further purified using GC Spin tips (GL-Tip, Titansphere, GL Sciences Inc, Japan) as previously described in [80].

### 2.12. NanoLC-MS/MS protein identification and quantification

Samples were resuspended in 35 µl of 10% ACN, 0.1% TFA in HPLC-grade water. For immunoprecipitation experiments, single run MS analysis was performed. For total proteome and phosphoproteome analysis, each sample was injected in technical triplicates. For each run, 5 µl were injected on a nanoRSLC-Q Exactive PLUS (RSLC Ultimate 3000, (Thermo Scientific, Waltham, MA, USA)). Peptides were loaded onto a µ-precolumn (Acclaim PepMap 100 C18, cartridge, 300 µm i.d.×5 mm, 5 µm (Thermo Scientific)), and then separated on a 50 cm reversed-phase liquid chromatographic column (0.075 mm ID, Acclaim PepMap 100, C18, 2 µm (Thermo Scientific)). Chromatography solvents were A (0.1% formic acid in water) and B (80% acetonitrile with 0.08% formic acid). Peptides were eluted from the column using a 120 minute-gradient from 5% to 40%, followed by 5 min at 80% and 20 min of re-equilibration at 5% B before next injection. One blank sample was run between each replicate to prevent sample carryover. Peptides eluting from the column were analysed by data-dependent MS/MS, using top-10 acquisition method, and fragmented using higher-energy collisional dissociation (HCD). The instrument's settings were as follows: resolution was set to 70 000 for MS scans and 17 500 for the data-dependent MS/MS scans to increase speed; MS AGC target was set to 3.10^6^ counts with maximum injection time set to 60 ms, while MS/MS AGC target was set to 1.10^5^ with maximum injection time set to 120 ms; MS scan range was from 400 to 2000 *m/z*. Dynamic exclusion was set to 30 s. The MS files corresponding to the interactome analysis and to the phosphoproteome and the total proteome analysis were processed differently. For interactome analysis, MS files were searched with the MaxQuant software v. 1.6.6 using Andromeda search engine against Andromeda search engine against the database of *Mus musculus* from swissprot 07/2017 and *Plasmodium berghei* ANKA from PlasmoDB (v. 37). To search for parent mass and fragment ions, a mass deviation was set at 3 ppm and 20 ppm respectively. The minimum peptide length was set to 7 amino acids and strict specificity for trypsin cleavage was required, allowing up to two missed cleavage sites. Carbamidomethylation (Cys) was set as fixed modification, whereas oxidation (Met) and N-term acetylation were set as variable modifications. The false discovery rates (FDRs) at the protein and peptide level were set to 1%. Scores were calculated in MaxQuant as described previously [81]. Match between runs was allowed. Proteins were quantified according to the MaxQuant label-free algorithm using LFQ intensities; protein quantification was obtained using at least 2 peptides per protein. The reverse and common contaminants hits were removed from MaxQuant output.

Statistical and bioinformatic analysis were performed with Perseus software (v. 1.6.14), freely available at https://maxquant.net/perseus/ [82]. For statistical comparison we set two groups: *Plasmodium berghei* parental (WT PBANKA GFP) and KO. Each group contained three biological replicates. We then filtered the data to keep only proteins with at least three valid values in at least one group. Next, the data were imputed to fill missing data points by creating a Gaussian distribution of random numbers with a standard deviation of 33% relative to the standard deviation of the measured values and 1.8 standard deviation downshift of the mean to simulate the distribution of low signal values. We performed a *t*-test represented on a volcano plot, FDR < 0.01, S0 = 1.

For proteomic and phosphoproteomic analysis, the MS files were processed with the MaxQuant software v. 1.5.8.3 and searched against the same database and the same settings as above, except for the following. Two distinct parameter groups were used for the search: phosphorylation (Ser, Thr, Tyr) were set as additional variable modifications only for phosphoproteomics data [81].

Statistical and bioinformatic analysis, including heatmaps, profile plots and clustering, were performed with Perseus software (v. 1.6.2.3 for phosphoproteomics analysis) freely available at https://maxquant.net/perseus/ [82]. For statistical comparison two groups were set: *P*. *berghei* parental (WT PBANKA GFP) and KO. Each group contained four biological replicates and each sample was run in technical triplicates. The phosphopeptides output table were used for phosphopeptide analysis. The table was expanded to separate individual phosphosites, murine proteins were excluded, and the *P. berghei* distribution of phosphosites was normalized using the option ‘with adjustment’. We kept phosphosites identified in all four replicates in at least one group (KO versus WT). Phosphosites with a phosphorylation localization probability less than 0.75 were excluded. Missing values were imputed using width = 0.15 and down-shift = 3. The significantly altered phosphosites (*t*-test S0 = 0.1, FDR = 0.05) were represented in a volcano plot. The protein groups output table was used for total proteome analysis, murine proteins were also excluded. We kept only proteins three replicates out of four in at least on group (WT and KO). Missing values were imputed as above.

The mass spectrometry proteomics data have been deposited to the ProteomeXchange Consortium via the PRIDE [83] partner repository with the dataset identifier PXD030389.

## 3. Results

### 3.1. *In silico* analysis revealed that *P. berghei* LRR1 possesses 11 LRR domains and adopts a typical horseshoe-like structure

To identify Leucine Rich Repeat protein 1 (LRR1) in *P. berghei*, we performed an OrthoMCL analysis (https://orthomcl.org/orthomcl/app) of *P. falciparum* LRR1 (PfLRR1, PF3D7_1032800), leading to the identification of PBANKA_0516600 (PbLRR1, orthomcl group: OG5_127611). PbLRR1 is encoded by a 4-exon gene and is composed of 309 amino acids, with a calculated molecular weight of 36.5 kDa (ProtParam analysis, https://web.expasy.org/protparam/).

**Figure 1.**
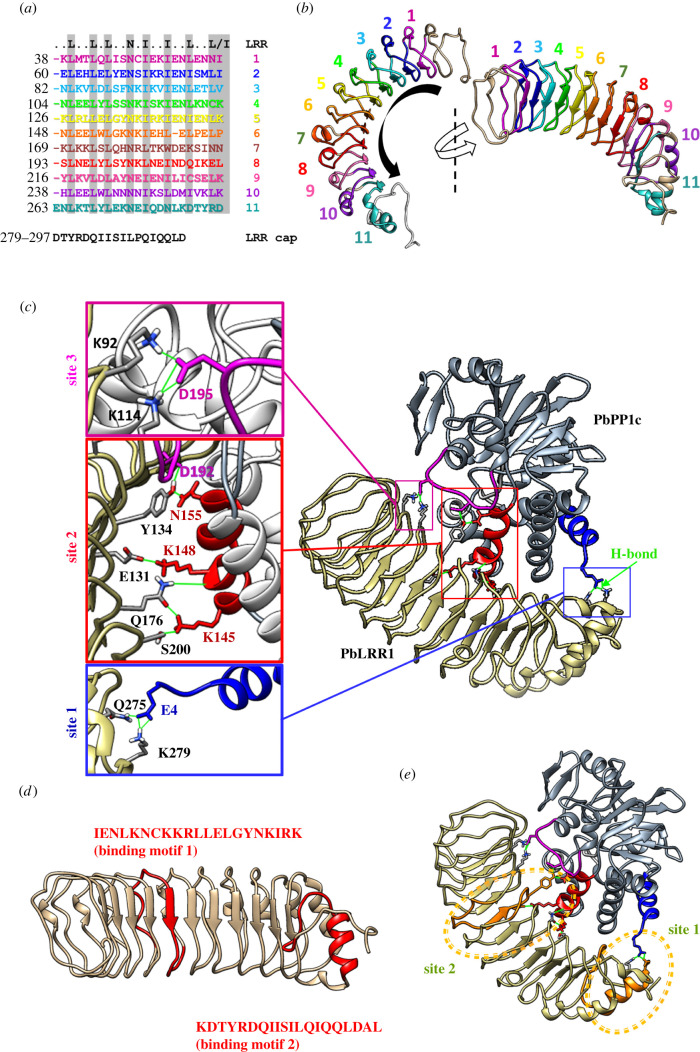
*Plasmodium berghei* Leucine-Rich-Repeat 1 (PbLRR1) LRR domains and structural model. (*a*) PbLRR1 schematic domain composition showing the 11 predicted Leucine Rich Repeat (LRR) domains fitting the **LXXLXLXXNXIXXIXXLXXL/I** consensus sequence. Is also represented the LRR cap domain. All predictions were made using Prosite software (). Numbers at the left side indicate the amino acid position at the beginning of each domain. (*b*) PbLRR1 tertiary structure predicted using I-TASSER and further annotated with Chimera 1.14. Each LRR domain is highlighted with the same colour code used in panel A. This panel shows that PbLRR1 folds into a typical horseshoe shape. (*c*) PbLRR1/PbPP1c in silico docking analysis. The model was built using HADDOCK 2.2 server (https://milou.science.uu.nl/services/HADDOCK2.2/haddock.php) and the complex displaying the most representative binding mode was kept for further analysis and annotation in Chimera 1.14. The three binding sites are emphasized, as well as the main hydrogen bonds involved in the formation of these sites. (*d,e*) PbLRR1 sequences corresponding to the PfLRR1 peptides described as able to disrupt PfLRR1/PfPP1c complex by Pierrot *et al.* [30] are highlighted in red in the PbLRR1 three-dimensional structure model (*d*) and then placed on the PbLRR1/PbPP1 complex *in silico* model (highlighted within orange circles (*e*)). Those binding analyses were carried out using Chimera 1.14.

Protein domain analysis (https://prosite.expasy.org/) revealed the presence of 11 Leucine Rich Repeat (or LRR) domains. LRR domains are formed from tandem arrays following the consensus sequence LXXLXLXXNXIXXIXXLXXL/I where LXXLXL constitutes a β-strand structure and XXNXIXXIXXLXXL/I is an α-turn domain (figure 1*a,b*; electronic supplementary material, figure S1A) LRR domains are known to be involved in various processes such as bacterial pathogenesis or plant immune response through macromolecular interactions [47,84,85]. As seen with other LRR domain-containing proteins, PbLRR1 sequence examination revealed the presence of a carboxy-terminal cap domain following the consensus sequence: **Y**RxxφxxxφPxφxxL**D** (amino acids 281 to 297, φ: hydrophobic residues) (figure 1*a*; electronic supplementary material, figure S1A [86]) crucial for the folding of those proteins. Using the I-tasser server, we generated a model of the three-dimensional (3D) structure of PbLRR1. The proposed model adopts a curved horseshoes structure typical of the LRR protein family, with 11 parallel β-strands located on the concave side and an α-helices on the convex side (figure 1*b*).

### 3.2. *In silico* docking analysis revealed an interaction of PbLRR1 to PbPP1c via 3 binding sites

Having previously established the presence of the PP1c/LRR1 complex in *Plasmodium* [23,32], we set out to gain more insights of the interaction at the molecular level by performing an *in-silico* docking analysis using a PbPP1c in silico model (https://swissmodel.expasy.org/repository/uniprot/A0A509AL46). This model has been built based on a crystal structure of the rat PP1c complexed with mouse inhibitor-2 protein (SMTL ID: 2o8a.1). PbPP1c shares 85.57% sequence identity with rat PP1c, and the QMEAN of the model (https://pubmed.ncbi.nlm.nih.gov/17932912/) is about 0.90, indicating that the folding of the PbPP1c model is nearly identical to that of mammalian PP1c (figure 1; electronic supplementary material, table S2).

Using predicted tertiary structures of both PbPP1c and PbLRR1, 156 putative interaction models were generated and grouped into eleven clusters using HADDOCK 2.2 server (https://milou.science.uu.nl/services/HADDOCK2.2/haddock.php). The binding site analysis was performed in UCSF Chimera 1.14 on the five clusters that showed the most common binding pattern (see 'Materials and methods'). This analysis indicated the presence of three binding sites between the two proteins, as highlighted by Heroes *et al.* with HsSDS22 and HsPP1c (figure 1*c*) [47]. Binding site 1 involves the carboxy-terminal end of PbLRR1 (both the cap and the last LRR domain) and PbPP1c α-helix1 (key binding residues: E4 in PbPP1c and Q275 & K279 in PbLRR1). Binding site 2 involves LRR domains 5, 7 and 8 located at the center of PbLRR1 and PbPP1c α-helix 6 (key binding residues: K145, K148 & D152 in PbPP1c and Y134 & K156 in PbLRR1). Binding site 3 involves the amino-terminus of PbLRR1 and the PbPP1c *α*7–α8 loop (key binding residues: D192 & D195 in PbPP1c and R28, S70, K92 or K114 in PbLRR1 (variable depending on models, see 'Materials and methods' for more detailed information). Interestingly, binding sites 1 and 2 seemingly rely on well-conserved interactions. Overall, these results are in perfect accordance with a previous study where we demonstrated that peptides derived from PfLRR1 (78.1% identity with PbLRR1, electronic supplementary material, figure S1A) comprising binding sites 1 and 2 could interact with PfPP1c and alter *P. falciparum* development *in vitro* when coupled to penetrating peptide sequences (see the peptides with orthologous sequences in PbLRR1 (figure 1*d*) and integrated to the PbLRR1/PbPP1c complex on figure 1*e* [30]. This strongly suggests that binding sites 1 and 2 may be the main interaction sites between LRR1 and PP1c.

### 3.3. PbLRR1 is expressed in asexual and sexual stages

To localize PbLRR1 expression throughout *P. berghei* development, an AID-HA-tagged transgenic parasite line was generated using a single homologous recombination strategy to the OsTIR1 expressing pG230 *P. berghei* ANKA line ([68], electronic supplementary material, figure S2A). Correct integration of the tag and tagged-protein expression were confirmed by integration PCR and western blot analysis (electronic supplementary material, figures S2B and S2C).

**Figure 2.**
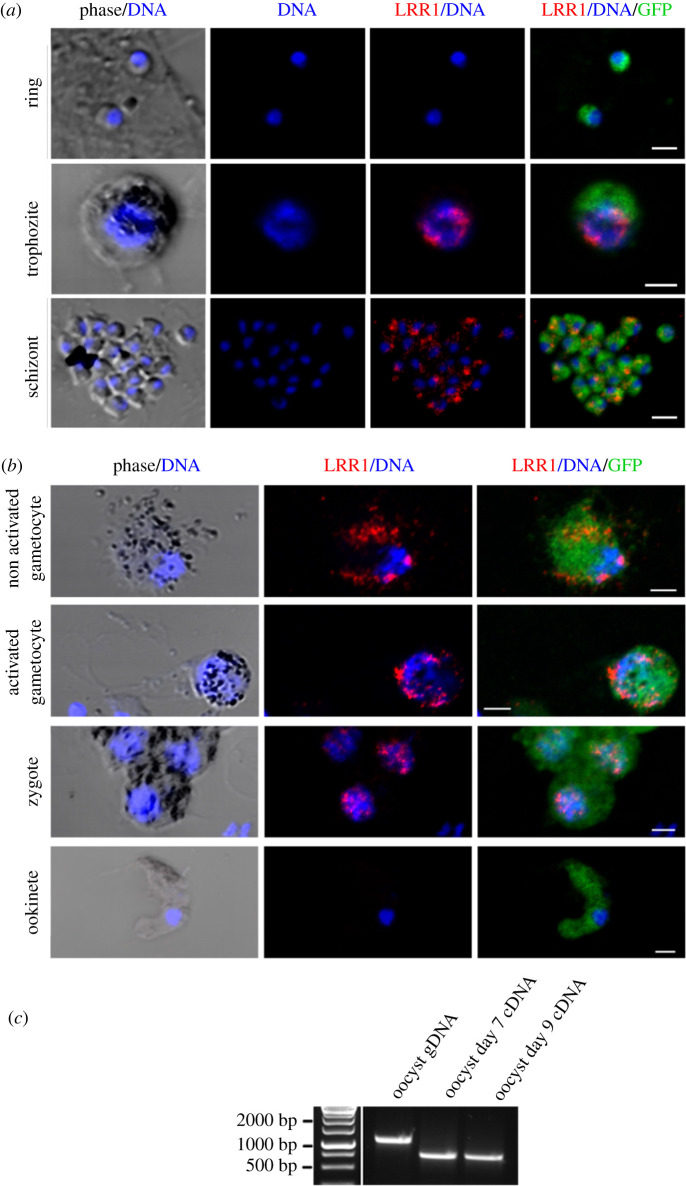
PbLRR1 expression and sub-cellular localization during *Plasmodium* life cycle. Confocal microscopy images of PbLRR1-AID-3HA (anti-HA mAb, red) expressing parasites in (*a*) rings, trophozoites, schizont, and in (*b*) non activated and activated gametocytes (30 min), zygotes (4 h post fertilization) and ookinetes (24 h post fertilization). GFP Parasites (green) nuclei are stained with DAPI (blue). Scale bar, 10 µm. (*c*) RT-PCR confirming the expression of *Pbrr1* mRNA in day 7 and day 9 oocysts (Pr1-Pr2, electronic supplementary material, table S1).

PbLRR1 expression and localization over the parasite life cycle was monitored by immunofluorescence assays using anti-HA antibodies. During the asexual development cycle in the blood, PbLRR1 was undetectable at ring stage but was found expressed in trophozoites at the periphery of the nucleus, and in schizonts as a cytosolic punctuated pattern (figure 2*a*). PbLRR1 was also observed as cytosolic and perinuclear dots in non-activated gametocytes. Its expression was also detected in activated gametocytes and zygotes (4 h) exhibiting a punctuated perinuclear pattern. By contrast, PbLRR1 was not detectable in ookinetes (figure 2*b*). These observations are consistent with the *PbLRR1* transcription profile extracted from *P. berghei* global gene expression analysis [87], showing that *pblrr1* is expressed at its highest level in trophozoite and gametocyte stages but is extremely low in ookinetes (electronic supplementary material, figure S2D).

### 3.4. The PbPP1/PbLRR1 complex is detected in both asexual and sexual stages

**Figure 3.**
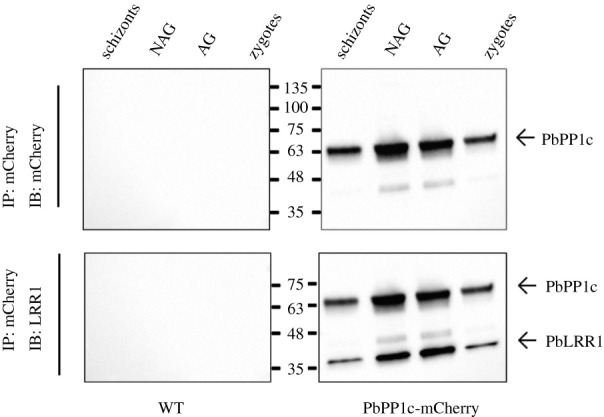
Immunoprecipitation assay of the PbLRR1/PbPP1 complex in schizonts, non-activated and activated gametocytes and zygotes. Investigation of the presence of the PbLRR1/PbPP1c complex during various stages of *P. berghei* development. Protein extracts from schizont, non-activated gametocyte (NAG), activated gametocyte (AG) and zygotes stages expressing mCherry-tagged PbPP1c were immunoprecipitated using anti-RFP antibodies followed by immunoblotting with the same Ab (upper panels) and anti-PfLRR1 antibodies (lower panels). Immunoblots were re-probed after several washings and without stripping.

In a previous study aiming to identify PbPP1c interacting partners during asexual development, we demonstrated that PbLRR1 belongs to the PbPP1c interacting network in schizonts [31,32]. Here, using an immunoprecipitation approach, we deepened the analysis and examined the presence of the complex at additional stages of *P. berghei* sexual development. We analysed eluates immunoprecipitated from a PbPP1c-mCherry line (previously generated in [31]) with a PfLRR1 polyclonal serum [23]. As shown in figure 3, the immunoblot analysis confirmed the detection of the PbPP1c/PbLRR1 complex in schizonts and showed its presence in gametocytes, activated gametocytes and zygotes (figure 3; electronic supplementary material, figure S5), suggesting a potential role in sexual development of the parasite.

### 3.5. PbLRR1 regulates XePP1c activity

**Figure 4.**
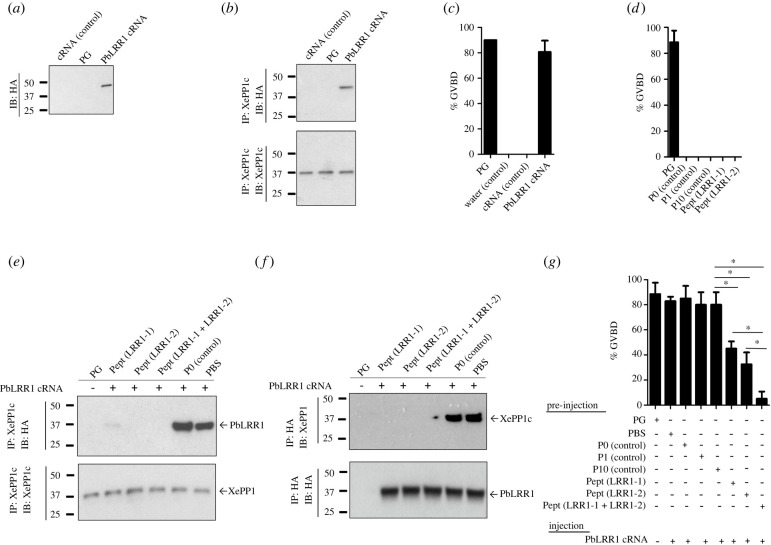
PbLRR1 functional analysis using *Xenopus* oocytes model. (*a*) PbLRR1 is expressed in *Xenopus* oocytes following the micro-injection of its cRNA. Immunoblot analysis of extracts were prepared from micro-injected oocytes with either PbLRR1 cRNA (60 ng in 60 nl, lane 3) or control cRNA (60 ng in 60nl, irrelevant protein, lane 1), or treated by progesterone (4 µg ml^−1^, PG, lane 2) using an anti-HA mAb. (*b*) Interaction of PbLRR1 with Xenopus PP1 (XePP1c). Extracts prepared from *Xenopus* oocytes previously micro-injected with either PbLRR1 cRNA (60 ng in 60nl, lane 3), or control cRNA (60 ng in 60nl, irrelevant protein, lane 1) or treated by a progesterone (4 µg ml^−1^, PG, lane 2) were immunoprecipitated using an anti-PP1 mAb followed by an immunoblotting using anti-HA mAb (upper panel) and anti-PP1 mAb (lower panel). (*c*) PbLRR1 can regulate PP1 activity. The appearance of GVBD induced by the micro-injection of PbLRR1 cRNA (60 ng in 60nl, lane 4), irrelevant control protein (60 ng in 60 nl, lane 3, negative control), water for the injection control (60 nl, lane 2) or after a progesterone treatment (4 µg ml^−1^) for the positive GVBD control (PG, lane 1), was monitored after 15 h. Values are presented as mean percentages and sem (error bars). Each experiment was performed using a set of 20 oocytes and repeated on three animals. (*d*) Percentage of GVBD induced after the micro-injection in oocyte of the peptide LRR1-1 (**VKKKKIKAEIKI**IENLQNCKKLRLLELGYNKIRM), LRR1-2 (**VKKKKIKAEIKI**ENYRKTIIHILPQLKQLDAL), the control peptide P0 (**VKKKKIKAEIKI**), the control peptide P1 (derived from *Plasmodium* inhibitor 2 PP1 interacting domain ((**VKKKKIKREI**KKTISWKKTISW)) and its mutated version P10 (**VKKKKIKREI**KKTASAKKTASA) or with progesterone (PG, as positive control). Each experiment was performed using a set of 8 oocytes and repeated on three animals. (*e*) Disruption of the PbLRR1/XePP1c interaction after the injections of peptides derived from the PfLRR1 sequence. Oocyte extracts were micro-injected with peptide LRR1-1 (lane 2), LRR1-2 (lane 3), LRR1-1 and LRR1-2 (lane 4), the control peptide P0 (lane 5), or with PBS (lane 6), 1-hour prior PbLRR1 cRNA. Then, the extracts were immunoprecipitated with anti-PP1 mAb and subjected to immunodetection using an anti-HA mAb (upper panel) and anti-PP1 mAb (lower panel). Each experiment was performed using a set of 20 oocytes and repeated on two animals. (*f*) The same oocyte extracts were immunoprecipitated with an anti-HA and subjected to immunodetection using anti-PP1 mAb (upper panel) and anti-HA mAb (lower panel). (*g*) The disruption of the PbLRR1/XePP1c complex affect PbLRR1 ability to induce a GVBD. Percentage of GVDB induced by the injection of PbLRR1 cRNA in *Xenopus* oocytes after the pre-injection of peptides derived from the sequence of PfLRR1 or Pf inhibitor-2 (P1 and P10, used as negative control) as described in (*d*). The percentages of GVBD induction after peptide pre-injection are: Peptide LRR1-1: 45%, Peptide LRR1-2: 32.50%, Peptides LRR1-1 and LRR1-2: 5.2%, PBS: 85%, Peptide control P0: 85%, Peptide control P1: 80%, Peptide control P10: 80%. *: statistically significant *p* < 0.05 (Mann–Whitney). Each experiment was performed using a set of 10 oocytes and repeated on three animals.

Having demonstrated that PbLRR1 and PbPP1c form a complex in both sexual and asexual stages of *P. berghei* development, we next analysed PbLRR1 ability to regulate PP1c activity. To do so, we took advantage of the *Xenopus* oocyte model which has been previously demonstrated as a potent tool for the functional study of PP1c regulators [24,28,32,33], including PfLRR1 [23]. This system is based on PP1c conservation across species (up to 85% identity between the PP1c proteins of *Xenopus laevis* and *P. falciparum* (electronic supplementary material, figure S1C)), and on the observation that inhibition of PP1c in *Xenopus* oocyte triggers a germinal vesicle breakdown (GVBD). GVBD is easily observable by the appearance of a white maturation spot at the animal hemisphere of the oocyte [88]. When expressed in *Xenopus* oocyte upon microinjection of its complementary RNA (figure 4*a*), PbLRR1 displayed the ability to both interact with *Xenopus* PP1c (figure 4*b*) and to induce GVBD in up to 80% of oocytes (figure 4*c*), similarly to progesterone used as a positive control. To confirm the specificity of those observations, additional experiments were carried out using competing peptides derived from LRR1 sequence. In earlier studies, we presented the proof-of-concept that peptides derived from domains involved in the interaction with PP1c could be used to disrupt holoenzyme formation and modify PP1 activity [24,28]. Recently, a screening of the *P. falciparum* LRR1 sequence by Pepscan led to the identification of two PfPP1c binding motifs: 1: IENLQNCKKLRLLELGYNKIRM and 2: ENYRKTIIHILPQLKQLDAL [30]. Further, these two peptides coupled to a N-term penetrating sequences (Pept LRR1-1: **VKKKKIKAEIKI**IENLKNCKKLRLLELGYNKIRK; Pept LRR1-2: **VKKKKIKAEIKI**KDTYRDQIISILPQIQQLDAL) were shown to be able to interact with PP1c and to affect *P. falciparum* growth *in vitro* [30]. Interestingly, the corresponding sequences in *P. berghei* (1: IENLKNCKKLRLLELGYNKIRK and 2: KDTYRDQIISILPQIQQLDAL) are present within the interaction site 1 and 2 identified in our *in silico* analysis (figure 1*d,e* and electronic supplementary material, figure S1). Taking advantage of LRR1 sequence conservation across species (electronic supplementary material, figure S1), those peptides were tested in the *Xenopus* oocyte model for their ability to disrupt the PbLRR1/XePP1c complex and thereby to prevent PbLRR1 regulation of XePP1c. To this end, we first established the absence of any effect of the peptide alone on PP1 activity (figure 4*d*). Then, the peptides were pre-injected alone or together in the oocyte one hour prior the injection of the *Pblrr1* cRNA. The presence of the PbLRR1/XePP1c complex was tested by an immunoprecipitation assay followed by a western-blot analysis. The appearance of GVBD was observed as described above. As presented in figure 4*e,f*, the pre-injection of LRR1.1 or LRR1.2 peptide, interfered with the formation of the complex. The PbLRR1/XePP1c interaction was totally disrupted using peptide LRR1.2, and only a faint residual band of PbLRR1 was detected after LRR1.1 injection (figure 4*e*). No interaction was observed when both peptides were pre-injected together. Neither the pre-injection of the control peptide (P0: VKKKKIKAEI) nor PBS interfered with the complex formation (figure 4*e,f*).

At the functional level, the GVBD induction was strongly reduced when peptides LRR1.1 and LRR1.2 were pre-injected separately or together. To test the specificity of the observed effect, additional experiments were carried out using the control peptide P0 and a peptide derived from the Pf inhibitor-2 PP1 interacting motif: KKTISW. This peptide referred as P1 (**VKKKKIKREI**KKTISWKKTISW) has been previously described as able to disrupt the Pf inhibitor2/XePP1 complex [24]. No significant effect was observed when PBS, P1 or its mutated version P10 (**VKKKKIKREI**KKTASAKKTASA) were pre-injected (figure 4*g*).

Altogether, these observations strongly suggest that PbLRR1 can inhibit PP1c activity, in a similar fashion to its *P. falciparum* orthologue [23]. The peptide interference experiment also confirmed the importance of the PbLRR1 interaction sites 1 and 2 identified in the *in silico* analysis. It is also interesting to note that the pre-injection of both peptides drastically reduced the GVBD induction when compared to the pre-injection of the peptides alone. This suggests that the coordinated function of both sites could be essential to the complex formation and the proper establishment of LRR1 regulatory activity.

### 3.6. PbLRR1 is involved in oocyst development

**Figure 5.**
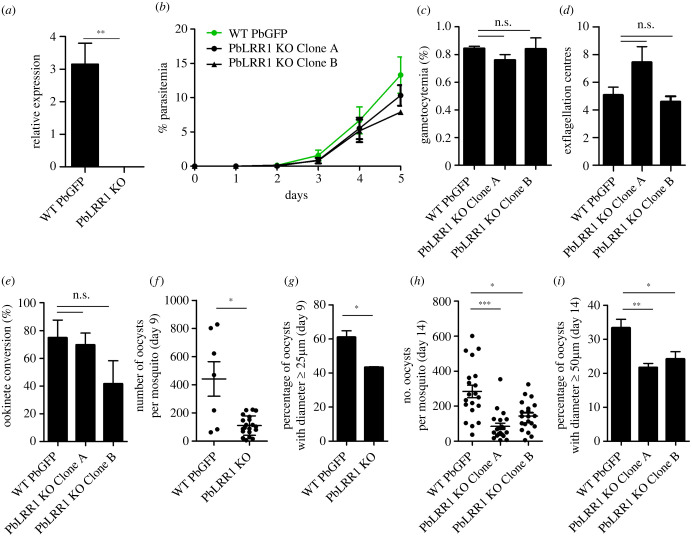
Phenotypic analysis of PbLRR1-depleted (PbLRR1 KO) versus wild-type (WT PbGFP) parasites. (*a*) *pblrr1* mRNA levels in blood stage parasites assessed by RT-qPCR. Values represent mean *pblrr1* mRNA levels relative to those of the houskeeping gene *arginyl-t RNA ligase* (PBANKA_1434200). (*b*) Asexual blood stage parasite development. (*c*) Gametocyte development. (*d*) Male exflagellation. Values represent mean exflagellation centres per field at 15 min post activation (10 fields per experiment). (*e*) Ookinete conversion. (*f*) Oocyst numbers at 9 days post mosquito infection (*n* = 10). (*g*) Percentage of oocysts with diameters ≥ 25 µm at 9 days post mosquito infection. Based on measurements of 611 oocysts from 3 different mosquitoes (pbGFP), and 464 oocysts from 3 different mosquitoes (PbLRR1KO). (*h*) Oocysts numbers at 14 days post mosquito infection (*n* = 20). (*i*) Percentage of oocysts with diameters ≥ 50 µm at 14 days post mosquito infection. Based on measurements of 3094 oocysts from 11 different mosquitoes (pbGFP), and 1811 oocysts from 20 different mosquitoes (PbLRR1KO). ns: not statistically significant; *: statistically significant *p* < 0.05; **: statistically significant *p* < 0.01); ***: statistically significant *p* < 0.0001) (Mann–Whitney).

Unlike the suggested essential role of LRR1 in blood stage of *P. falciparum* [58], recent *P. berghei* high-throughput functional analysis of disruption phenotypes found LRR1 to be dispensable for the parasite asexual development cycle [66]. To confirm these results and deepen the analysis of PbLRR1 function throughout the life cycle, notably in the mosquito stages, we took advantage of the readily available PlasmoGEM plasmid (PbGEM-232294) to generate two independent knock-out (KO) lines in the PbGFP ANKA strain (electronic supplementary material, figure S3A [67]). After pyrimethamine selection of integrants and cloning by limiting dilution, the deletion of *pblrr1* and integration of the *dhfr/ts* cassette was confirmed using diagnostic PCR (electronic supplementary material, figure S3B and S3C). Additionally, the absence of transcript was confirmed by quantitative RT-PCR (qRT-PCR) (figure 5*a* and electronic supplementary material, figure S3D). No variation in the number of schizont nuclei nor obvious growth rate variation was observed in the KO lines during blood-stage development (figure 5*b* and electronic supplementary material, figure S6A), consistent with the earlier functional study [66]. These observations demonstrate a redundant role of LRR1 in this part of the life cycle. Deletion of PbLRR1 did not significantly affect gametocytemia (figure 5*c*), exflagellation (male gamete formation) (figure 5*d*), or ookinete conversion (figure 5*e*), indicating a redundant role of PbLRR1 in gametocytogenesis, gametogenesis, fertilization and ookinete formation. We next performed transmission experiments and examined oocyst formation and maturation in the mosquito. A significant decrease ( approx. 70%) in the number of oocysts was observed in LRR1 KO parasites at day 9 post infection (figure 5*f*), and oocysts at this stage of development were smaller in size (figure 5*g*). Similarly, at two weeks post infection, LRR1KO oocyst numbers were significantly lower (approx. 60%, figure 5*h*) and at this stage of development too, oocyst sizes were significantly reduced (figure 5*i*). These phenotypes were observed in two independent LRR1KO clones, indicating that they are not the result of clonal variation but reflect a genuine effect of LRR1 depletion. Despite the overall smaller oocyst numbers and sizes in the PbLRR1KO lines, a substantial proportion of oocysts completed development to sporozoites and no significant effects on salivary gland colonization were observed (Exp 1: PbGFP: 62 salivary gland sporozoites per oocyst; LRR1-KO clone A: 66 sg spz/ooc; LRR1-KO clone B: 21 sg spz/ooc, n = 22 _Exp 2: PbGFP: 294 sg spz/ooc; clone A: 352 sg/ooc). Additionally, sporozoites were infective in both *in vitro* hepatocyte cultures (electronic supplementary material, figure S6C) and in mice (electronic supplementary material, figure S6D), albeit with a slightly longer prepatent period (electronic supplementary material, figure S6E). Overall, these results indicate that PbLRR1 depletion impacts the pre-sporulation stage of oocyst development.

### 3.7. PbLRR1 interactome during sexual development

**Figure 6.**
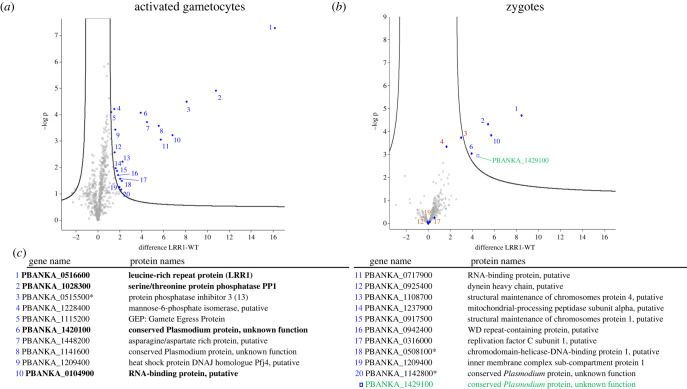
PbLRR1 interactome analysis. (*a*) Volcano plot representation of the outcome of the PbLRR1 interactome study carried out in activated gametocytes. The proteins highlighted in blue were identified as PbLRR1 interacting partners (*t*-test, FDR < 0.01, S0 = 1). (*b*) Volcano plot representation of the outcome of the PbLRR1 interactome study carried out in zygotes. The proteins highlighted in blue were identified as PbLRR1 interacting partners. The protein highlighted in green was specifically identified at that stage as a PbLRR1 interacting partners (*t*-test, FDR < 0.01, S0 = 1). The proteins highlighted in brown were identified as partners in the activated gametocytes analysis but not in zygotes. (*c*) List of PbLRR1 interacting partners with names and PlasmoDB accession numbers. The proteins highlighted in bold were identify as PbLRR1 partners in both analyses. The proteins followed by an asterisk were later identified in the phospho-proteomic analysis (figure 7).

To explore in more detail the functional pathways involving PbLRR1 during *Plasmodium* sexual development, we performed a PbLRR1 immunoprecipitation, followed by a mass-spectrometry identification of potential partners (IP-MS). We focused our analysis on the activated gametocyte and zygote stages during which PbLRR1 was found in complex with PbPP1c and highly expressed (figure 2). After several unsuccessful attempts using the HA tagged line, we generated a knock-in line where the endogenous PbLRR1 is fused to an m-Cherry tag (electronic supplementary material, figure S4). For IP-MS experiments, we performed three independent biological replicates and used the WT parental line as a control for contaminant interactions. As shown in electronic supplementary material, table S3A and S3B, the quality of the IP-MS was confirmed by the identification of the bait. After statistical analysis (*t*-test, FDR < 0.01, S0 = 1), a total of 20 proteins (activated gametocytes analysis) and 5 proteins (zygotes analysis) were found to be enriched in the PbLRR1-mCherry IP. Hit proteins were ranked based on their q-value and represented on a volcano plot (figure 6*a,b*). The complete list of identified proteins is provided in electronic supplementary material, table S3A and S3B. Regarding the activated gametocytes analysis, PbPP1c and the PP1c protein inhibitor 3 (PbI3) were the most highly enriched proteins identified in the analysis, suggesting that both proteins may form a heterotrimeric complex with LRR1. Such a complex has already been described in yeast and mammalian cells and proved to be crucial for proper cell cycle progression [45,89–91].

Closer inspection led to the identification of two proteins belonging to the SMC (structural maintenance of chromosome) family: PbSMC1 and PbSMC4. SMC1 is known to belong to the cohesin multiproteic complex involved in sister chromatid adhesion and proper chromosome segregation [92]. In *P. berghei*, SMC4 has been recently described as essential for sexual proliferation and involved in oocysts size and development [93]. Additionally, we identified ISP1, a blood stage essential protein, which belongs to the inner membrane complex (IMC). The IMC is a compartment restricted to the *Apicomplexa* critical for parasite gliding motility [94] and cytokinesis [95]. Finally, we detected PbGEP (Gamete Egress Protein), a *Plasmodium* specific protein involved in gametes fertility [96].

Regarding the analysis carried out at the zygote stage, only 4 proteins were retrieved from the analysis. Three of them (PP1c, PBANKA_1420100, PBANKA_01014900) have already been identified in the activated gametocytes analysis. One protein (PBANKA_1429100) seems specific to that stage (figure 6*c*, highlighted in green). It is interesting to note that, although PbPP1c remains among the most enriched protein, PbI3 was not identified as a potential PbLRR1 interactor in the zygotes (although the protein was observed at the limit of the threshold of significance and, at the RNA level, is predicted to be highly expressed in ookinetes [87]). This suggests that the PP1c/LRR1/I3 heterotrimeric complex may be restricted to specific development stages in *P. berghei.* Collectively, these observations suggest that PbLRR1 retains some of the functions previously observed in other organisms (i.e. cell cycle progression/division), while also engaging in *Plasmodium-*specific features (i.e. early sporogony).

### 3.8. The impact of PbLRR1 depletion on protein phosphorylation in zygotes

Having established PbLRR1 ability to bind and regulate PbPP1c activity, and a role during early stages of sporogony, we next investigated in more details how the absence of LRR1 protein could affect the phosphorylation status of proteins during sexual development. Our main aim was to identify candidate proteins supporting the PbLRR1KO phenotype, and additionally to identify putative PbPP1c substrates whose phosphorylation can be impacted by the lack of LRR1/PP1 complex formation. To this end, we performed a quantitative mass spectrometry analysis of the proteome and the phosphoproteome of the parental and PbLRR1KO lines in zygotes, the latest sexual stage in which the PbLRR1 protein can be detected (figure 2). These experiments were carried out on four biological replicates, with three technical triplicates each. The whole proteome analysis led to the identification of 1138 *Plasmodium* proteins. As expected, PbLRR1 was not detected in the KO line (electronic supplementary material, table S3C). Regarding PbPP1c, it was detected in all samples with no variation of its expression suggesting that the absence of PbLRR1 does not influence the phosphatase abundance in zygotes.

**Figure 7.**
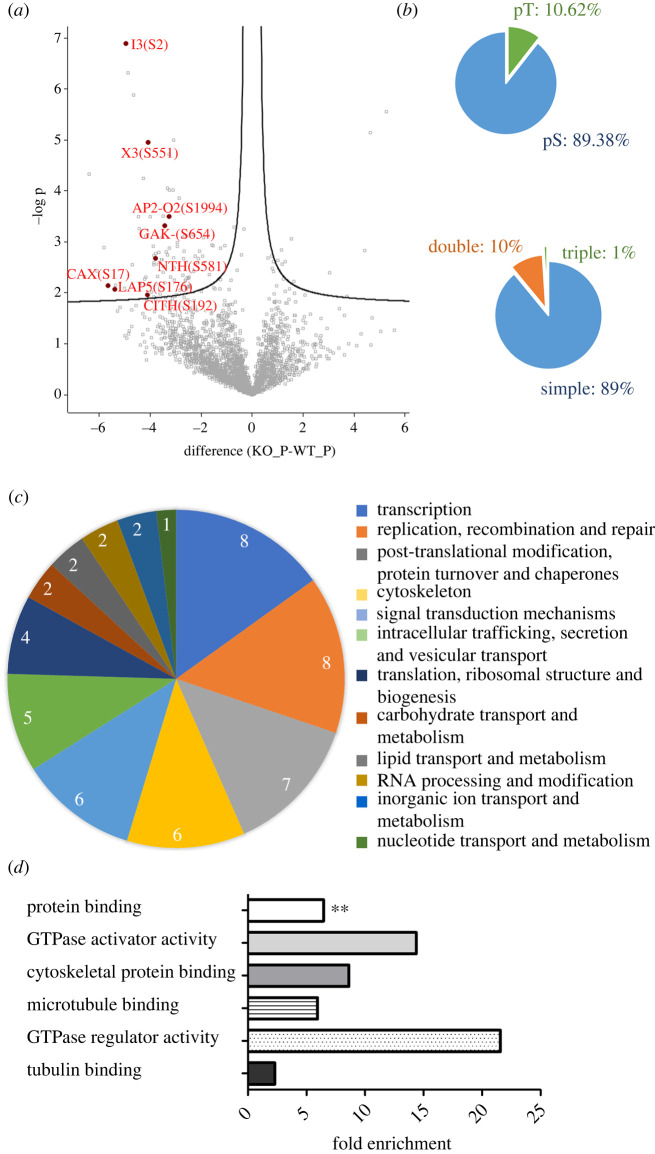
PbLRR1KO phospho-proteomic analysis. (*a*) Volcano plot representation of the outcome of the PbLRR1 KO phospho-proteomic analysis carried out in zygote. A selection of proteins carrying phosphosites with a significant differential phosphorylation status between the KO line and the parental line were highlighted in red (*t*-test S0 = 0.1, FDR = 0.05). Those proteins have been selected based of their interest in the study and/or their previous characterization in the sexual development of *Plasmodium berghei.* (*b*) Distribution of phosphoSer (pS), phospho-Thr (pT) residues and multiplicity of phosphosites detected in the analysis (*n* = 1138). (*c*) Functional annotation (based on the COG database) of proteins identified in the analysis with a differential phosphorylation status in the KO line versus parental line. (*d*) GO terms enrichment analysis. Fold enrichment was performed on the 100 proteins identified as hypophosphorylated in the PbLRR1 phosphoproteome. The *x*-axis represents the fold enrichment for the indicated biological function (hypergeometric test, Bonferroni correction **: statistically significant *p <* 0.01).

The phosphoproteomic analysis led to the identification of a total of 3217 phosphorylation sites (2647 *P. berghei* and 570 *Mus musculus* proteins). *P. berghei* phosphorylation sites were distributed over 1083 proteins. Among those, 112 sites, distributed over 100 proteins, were significantly different (Student *t* test, FDR = 0.05) with 99 sites being reduced and only 13 sites increased in the PbLRR1 knockout line. A total of 96 sites were changed by greater than 2-fold, with 90 exhibiting a greater than 2-fold decrease but only 6 exhibiting a greater than 2-fold increase in the PbLRR1 KO (figure 7*a,b*). We checked that these proteins, when detected, did not vary in the global proteome analysis. Indeed, integration of the proteomics and phosphoproteomics data allowed us to verify that the modulation observed in the phosphoproteome was linked to the phosphorylation itself rather than variations in protein abundance. Likewise, PbPP1c expression remains unchanged in the KO lines suggesting that the observed variations of phosphorylation levels must be related to a mislocalization and/or inadequate control of PP1c activity rather than to its absence.

When possible, the proteins whose phosphorylation significantly varied were classified based on their putative biological processes (COG database, [97]). The processes detected were diverse, spanning from transcription and RNA processing (25%), replication (20%), translation (10%), and post-translation modification (17.5%), to cytoskeleton/motility (15%), trafficking (12.5%) and signal translation (12.5%) (figure 7*c* and electronic supplementary material, table S3D). Next, we performed a GO enrichment analysis on the 100 dysregulated proteins and identified one molecular function related to protein binding (G0:0005515) to be significantly associated (23 proteins, Bonferroni adjusted *p* < 0.05 figure 7*d* and electronic supplementary material, table S3E).

Manual inspection of the data led to the identification of 7 hypophosphorylated proteins previously characterized in *Plasmodium* sexual development (figure 7*a*; data summarized in electronic supplementary material, table S4), with some showing specific features during oocyst development and/or maturation. For example, we identified PbGAK, a cyclin G-associated kinase, the knock-out of which has been shown to induce a strong reduction in oocyst production due to the absence of sporoblast formation and the development of unusually large oocysts [6]. Likewise, PbLAP5, a lectin-adhesive like protein localized in the crystalloids, is involved in oocyst development, with its deletion blocking sporozoite development [98,99]. Equally interesting was the observation that AP2-O2, a transcription factor involved in ookinete maturation, oocyst production and development [100] and CITH, a female gamete transcription repressor, were among the potential PP1/LRR1 substrates.

Most importantly, we observed a significant decrease in the phosphorylation of PbI3 (Serine 2) in the PbLRR1 KO line. This observation is of considerable interest as PbI3 has also been identified in the activated gametocytes PbLRR1 interactome (figure 6) and as a direct partner of PP1c in an earlier study [25]. This suggests that I3 may require a prior interaction with the LRR1/PP1c complex for a suitable control of its phosphorylation status. Two other hypophosphorylated proteins meet the same criteria; PBANKA_0508100 and PBANKA_1142800 (figures 6 and 7) which have not been characterized yet.

## 4. Discussion

In this study we investigated LRR1, a *Plasmodium* homologue of SDS22, one of the most ancient, ubiquitously expressed PP1c interactor involved in diverse cell development processes [37,41,44,45,101]. Unlike other interacting factors, SDS22 is known to be a structured protein [37], that does not interact with PP1 via intrinsically disordered regions (IDRs). In a recent crystal structure analysis, SDS22 was shown to interact via the concave side of its LRR-domains. The protein forms three bindings sites interacting with PP1c alpha helices 5 and 6 and the loop between alpha helices 7 and 8 [47]. Using protein domain and *in silico* docking analysis, we show that *P. bergh*ei LRR1 possesses 11 LRR domains flanked by a carboxy-terminal cap domain. The protein adopts the expected curved horseshoe tertiary structure and seems to interact with PP1c in a similar fashion as described in mammals [47]. Interestingly, our *in silico* model is in perfect agreement with a previous *P. falciparum* LRR1/PP1c interaction study, where PfLRR1-derived peptides comprising binding sites 1 and 2, were able to interact with PP1c and inhibit parasite development *in vitro* [30]. *In vivo*, we confirm the presence of the PbLRR1/PbPP1c complex at various stages of *P. berghei* development. We also confirm PbLRR1's capacity to interact and to inhibit PP1c function, like its *P. falciparum* counterpart [23]. Additionally, we demonstrated the contribution of the interaction sites 1 and 2 in the binding process and in LRR1 regulatory activity. Additional experiments will be required to confirm the involvement of the third binding site identified in our *in-silico* analysis.

To further study PbLRR1 function, we examined the impact of its deletion during *P. berghei* development. Unexpectedly, PbLRR1 is not refractory to deletion during blood stage development, unlike PfLRR1 that is predicted to be essential based on parasite fitness using piggyBac transposon insertions [58,66]. This allowed us to further study the PbLRR1 contribution to sexual, sporogonic, and liver stage parasite development. The functional redundancy of PbLRR1 for blood stage parasite development highlights an intriguing difference of LRR1 in regulating blood stage parasite PP1c between the rodent and human *Plasmodium* species. This cannot be explained at the genome level as those organisms only possess one copy of *lrr1* and no evident paralogs have been detected in *Plasmodium berghei*. Similarly, it is unlikely that a difference in LRR1 structure is accountable for this divergence because of the high degree of structural conservation of both proteins (electronic supplementary material, figure S1A). It is more likely that the role of LRR1 in *P. berghei* is less constrained, which could be due to the presence of additional regulators that are absent in *P. falciparum*. This requires further investigations.

Our analysis of the PbLRR1-depleted line development in the mosquito reveals a clear role for LRR1 during sporogony, that manifests by the production of overall fewer and smaller oocysts in the knockout lines. This oocyst-specific phenotype is fully consistent with findings from a recent genome-scale barcode study analysing the contribution of over 1300 *P. berghei* genes throughout the parasite life cycle. Using pools of gene knockout parasites, the study showed that the blood stage-to-midgut oocyst transition of LRR1-depleted parasites is significantly reduced [102]. This adverse effect is most likely directly caused by the absence of the protein in the oocyst. Although we could not assess LRR1 protein expression in oocysts, because indirect immunofluorescence on this life stage is notoriously difficult due to the presence of the oocyst capsule, its gene expression has been demonstrated in oocysts of *P. falciparum* [103,104], as well as in the other replicative stages of *P. berghei* [87,105–107] and has been confirmed in this study for *P. berghei* oocysts. Despite LRR1-KO oocysts being smaller, they display normal sporulation and levels of sporozoite production, indicating that the role of LRR1 is confined to the endomitotic phase of sporogony before cytokinesis. An adverse effect on mitosis and growth could also lead to a greater level of developmental arrest in early sporogony, explaining the smaller numbers of oocysts observed in mosquitoes on days 9 or 14 post-infection. This fits with the known roles of these PP1 interactors in cell cycle progression and stage completion [41,44,45,108]

By contrast to PbLRR1, PbPP1c is essential for blood-stage asexual parasite development and seems equally important during sexual development. A decrease in PbPP1c expression at the gametocyte stage led to drastic effects on the parasite development in the mosquito with a reduction in male exflagellation and ookinete conversion and no further oocyst or sporozoite production [109]. Depletion of PbLRR1, being an inhibitor of PbPP1c activity, is expected to have the opposite effect as it leads to hypo-phosphorylation of PP1 substrates, consistent with the different loss-of-function phenotypes of these two proteins.

In mammalian cells, the interaction site of SDS22 to PP1c has been localized remotely from other PP1 interactors, suggesting that SDS22 could be a member of multiprotein complexes of diverse PP1 holoenzymes [47]. To identify such complexes in *Plasmodium,* IP experiments of tagged PbLRR1 were performed at the activated gametocyte and zygote stages. This led to the identification of, respectively, 19 and 4 different proteins. Among those, we retrieved PP1c, confirming the presence of one or more PP1/LRR1 complex(es) in the parasite. In the activated gametocytes interactome, we also identified Inhibitor 3 (I3), a well-known PP1 regulator. From the available literature, SDS22/I3/PP1c heterotrimeric complexes have been described in various models. In yeast, this complex is essential to PP1 and SDS22 nuclear localization [89,90]. In mammalian cells, it is crucial for PP1 activation mediated by SDS22 and for PP1c transport to kinetochores where it can antagonize Aurora B and ensure a timely mitotic progression [45,91]. We also identified 2 proteins belonging to the SMC (Structural Maintenance of Chromosomes) family: PbSMC1 and PbSMC4. SMC1 belongs to the cohesin multiproteic complex involved in chromosome segregation [92]. PbSMC4 is of significant interest as it is important for sporogony. Indeed, the knock-down of this protein in gametocytes leads to the production of fewer and smaller oocysts [93]. Of note, PP1 and SMC4 both have been shown to be localized at the kinetochore [93,109]. PbLRR1 is likely to localize at the kinetochore in accordance with SDS22 protein location, and consistent with its observed nucleus-associated localization in *P. berghei* (figure 2). To deepen our understanding of LRR1 function during *Plasmodium* sexual development, a phosphoproteomic analysis was performed at the zygote stage and 113 phosphorylation sites (located on 100 unique proteins) were detected. Interestingly, some of these proteins had already been described as essential for *P. berghei* sexual development. This is the case with the transcription factor AP2-O2, which is hypophosphorylated in our KO line, and crucial for oocyst development in both *P. berghei* and *P. yoelii* [100,110]. PBANKA_0609500 (X3) was also detected as differentially phosphorylated in the phosphoproteome analysis. Although this protein has not been characterized yet, it belongs to the kinesin family. Kinesins are motor proteins key to motility and cell division. In *Plasmodium*, the deletion of one of its members, kinesin-8X, is detrimental to oocyst development [111]. All these findings thus correlate with a potential role of LRR1 in *Plasmodium* development in the mosquito, via the regulation of PP1.

Finally, the phosphoproteome analysis strongly supports the involvement of PP1c in the regulation of I3 phosphorylation status in PbLRR1 KO zygotes. Indeed, to the best of our knowledge, it is the first time that this protein, previously described in *Plasmodium* as a regulator of PP1 [25] has been found to be hypophosphorylated in the absence of LRR1. This strongly indicates that I3 may be a substrate of PP1c, and its correct phosphorylation level could be regulated by the LRR1/PP1c complex. Thus, I3 phosphorylation status may determine its ability to both interact with the complex and to regulate its activity as previously described in other organisms [45].

## Data Availability

**DNA sequences** PbLRR1: PBANKA_0516600 (PlasmoDB.org) XP_034420407.1 (geneBank) PbPP1**:** PBANKA_1028300 (PlasmoDB.org), XP_008624004.1 (geneBank) **Proteomic and Phospho-proteomic analysis** Proteomic and phospho-proteomic analysis that support the findings of this study have been deposited in the ProteomeXchange Consortium via the PRIDE [83] partner repository with the dataset identifier PXD030389. The data are provided in electronic supplementary material [112].
